# Micropropagation of Ginger (*Zingiber officinale* Roscoe) ‘Bentong’ and Evaluation of Its Secondary Metabolites and Antioxidant Activities Compared with the Conventionally Propagated Plant

**DOI:** 10.3390/plants10040630

**Published:** 2021-03-26

**Authors:** Nisar Ahmad Zahid, Hawa Z. E. Jaafar, Mansor Hakiman

**Affiliations:** 1 Department of Crop Science, Faculty of Agriculture, University Putra Malaysia, Serdang 43400 UPM, Selangor, Malaysia; znisarahmad15@gmail.com (N.A.Z.); hawazej@upm.edu.my (H.Z.E.J.); 2 Department of Horticulture, Faculty of Plant Sciences, Afghanistan National Agricultural Sciences and Technology University, Kandahar 3801, Afghanistan; 3 Laboratory of Sustainable Resources Management, Institute of Tropical Forestry and Forest Products, Universiti Putra Malaysia, Serdang 43400 UPM, Selangor, Malaysia

**Keywords:** *Zingiber officinale* Roscoe, micropropagation, surface sterilization, multiplication, rooting, phenolic acid, flavonoid

## Abstract

‘Bentong’ ginger is the most popular variety of *Zingiber officinale* in Malaysia. It is vegetatively propagated and requires a high proportion of rhizomes as starting planting materials. Besides, ginger vegetative propagation using its rhizomes is accompanied by several types of soil-borne diseases. Plant tissue culture techniques have been applied in many plant species to produce their disease-free planting materials. As ‘Bentong’ ginger is less known for its micropropagation, this study was conducted to investigate the effects of Clorox (5.25% sodium hypochlorite (NaOCl)) on explant surface sterilization, effects of plant growth regulators, and basal media on shoots’ multiplication and rooting. The secondary metabolites and antioxidant activities of the micropropagated plants were evaluated in comparison with conventionally propagated plants. Rhizome sprouted buds were effectively sterilized in 70% Clorox for 30 min by obtaining 75% contamination-free explants. Murashige and Skoog (MS) supplemented with 10 µM of zeatin was the suitable medium for shoot multiplication, which resulted in the highest number of shoots per explant (4.28). MS medium supplemented with 7.5 µM 1-naphthaleneacetic acid (NAA) resulted in the highest number of roots per plantlet. The in vitro-rooted plantlets were successfully acclimatized with a 95% survival rate in the ex vitro conditions. The phytochemical analysis showed that total phenolic acid and total flavonoid content and antioxidant activities of the micropropagated plants were not significantly different from the conventionally propagated plants of ‘Bentong’ ginger. In conclusion, the present study’s outcome can be adopted for large-scale propagation of disease-free planting materials of ‘Bentong’ ginger.

## 1. Introduction

Medicinal plants have long been used in the treatment of several diseases throughout the world. Ginger (*Zingiber officinale* Roscoe), a herbal plant from the Zingiberaceae family, is one of those plants. It has been used as spice and medicine for treating cancer [1]; cardiovascular disease [2]; diabetes [3]; and several other illnesses such as cold, nausea, asthma, and cough [4]. Owing to the global pandemic of COVID-19, ginger consumption gained more interest. It helped alleviate the severe symptoms of COVID-19 positive patients and reduced the recovery time in those patients [5,6]. The presence of a high polyphenols and flavonoids content in its leaves, stem, and rhizome has been defined as the critical factor for its pharmacological effects [7]. These polyphenols and flavonoids compounds are natural sources of antioxidants [8]. Ginger rhizome extract is a rich source of those antioxidants and, owing to its antimicrobial activity, it is also demonstrated as a natural candidate in food preservation [9]. Because of all these unique characteristics of ginger, its demand is highly increased in the world markets.

Ginger is an unfertile species that failed to set seed. Thus, it cannot be sexually propagated because its rhizomes are used for its vegetative propagation [10]. As rhizome is an economically exploited part of the plant, using a high proportion of ginger rhizomes as starting material for cultivating the plant in the next growing season negatively affects its supply in the market. Besides, most of the diseases such as bacterial shrivel (*Pseudomonas solanacearum*), leaf spot or blast (*Phyllosticta zingiberi* and *Pyricularia zingiberi*), delicate decay (*Pythium aphanidematum*), and yellowing of leaf (*Fusarium oxysporum*) are easily transmitted through vegetative reproduction by fragmentation of the rhizomes [11,12]. Furthermore, the bulkiness of ginger rhizomes as planting material makes it costly and laborious to handle. Therefore, in vitro propagation is a suitable alternative for the effective production of ginger.

The in vitro technique’s success largely depends on the aseptic culture establishment, shoot regeneration capacity, rooting, and acclimatization. Rhizome buds, which are often used as the source of explants in Zingiberaceae, have been proven to be more responsive. However, the initial establishment of contamination-free culture is difficult owing to the exposure of rhizomes to various soil pathogens [13,14]. These pathogens need to be eliminated by surface sterilization of the explants. To control the contamination by surface sterilization of the explant, the type and concentration of the disinfectants need to be carefully selected to prevent their noxious effect to plant tissues. Mercuric chloride (HgCl_2_) effectively reduced contamination in the rhizome sprouted buds explants of ginger [15], but it is toxic to either human or plant tissues [16,17]. Clorox bleach, containing 5.25% sodium hypochlorite (NaOCl), is an alternative safe explant surface sterilant. The effectiveness of Clorox depends on its optimized concentration according to different plant species and explant types [15,18,19].

On the other hand, shoot regeneration capacity depends on the formulation of culture media and plant growth regulators, mainly cytokinins. Murashige and Skoog (MS) medium [20] supplemented with 6-benzylaminopurine (BAP) is more commonly used for ginger shoot multiplication [14,21,22]. Apart from BAP, kinetin [23] and thidiazuron (TDZ) [24] also differently influenced shoot multiplication of different varieties of ginger. In addition, a developed root system is the crucial step in plant vegetative propagation, which ensures a high survival rate of micropropagated plantlets during the acclimatization stage [25]. In vitro root induction of ginger is enhanced by auxins supplementation in the culture medium [26,27]. Different types and concentrations of auxin varyingly affected in vitro root induction in different Zingiberaceae species [27,28]. Determining the optimum type and concentration of auxin can significantly enhance the in vitro root induction of ginger and, subsequently, facilitates acclimatization and successful establishment of the in vitro-raised plantlets in the field conditions.

The micropropagation of ‘Bentong’ ginger, a well-known variety of *Zingiber officinale* in Malaysia, has been less understood. Therefore, this study was carried out to establish a successful protocol for direct in vitro regeneration of ‘Bentong’ ginger. This study’s objectives were to determine Clorox’s effective concentration for explant surface sterilization of ‘Bentong’ ginger. The study also aimed to determine the best culture medium and the optimum type and concentration of cytokinins for shoots multiplication of ‘Bentong’ ginger and obtain the best type and concentration of auxin for the in vitro rooting of ‘Bentong’ ginger. The total phenolic acid and total flavonoid content and antioxidant activity of the micropropagated plants were also evaluated in this study compared with the conventionally propagated plants of ‘Bentong’ ginger.

## 2. Results

### 2.1. Micropropagation of ‘Bentong’ Ginger

#### 2.1.1. Explant Surface Sterilization of ‘Bentong’ Ginger

Increasing Clorox concentration from 30 to 70% significantly increased (*p* < 0.05) the aseptic culture percentage (Table 1). All explants were contaminated in the control treatment (without using Clorox). The treatments of 30 and 40% Clorox were not effective for eliminating the contaminations. They resulted in 41.25 and 43.75% axenic explants, respectively, and were not significantly different from the treatment of 50% Clorox, which resulted in 51.25% contamination-free explants. The treatment of 60% Clorox resulted in 57.50% aseptic explants, which was not significantly different from 50% Clorox. Using 70% Clorox, the contamination was significantly reduced and 75% aseptic explants were obtained.

**Figure 1:**
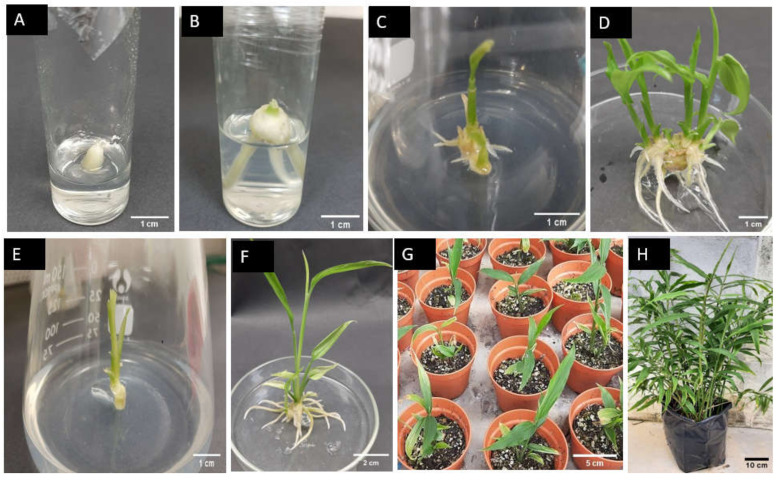
Micropropagation of ‘Bentong’ ginger. (**A**) Rhizome sprouted bud of ‘Bentong’ ginger used as explant for culture initiation; (**B**) aseptically established culture when surface sterilized with 70% Clorox after four weeks of inoculation; (**C**) in vitro-raised shoot used as explant for shoot multiplication (after 10 days of inoculation on MS medium supplemented with 10 µM zeatin); (**D**) multiple shoots produced in MS medium supplemented with 10 µM of zeatin after six weeks of inoculation; (**E**) in vitro-raised shoot used as explant for rooting; (**F**) in vitro-rooted plantlet derived from MS medium supplemented with 7.5 µM NAA after four weeks of culture; (**G**) acclimatized plantlets after three weeks of acclimatization; (**H**) in vitro-raised plant of ‘Bentong’ ginger after seven months of transplanting.

The explants’ survival rate was reduced by increasing the Clorox concentration up to 60–70%. Clorox at the concentration of 30–50% was not toxic to the explant tissues and 100% of the explants survived. Surface sterilization of explants with 60 and 70% Clorox resulted in a 91.48 and 83.60% survival rate, respectively. Owing to the 100% contamination, no explant survived in the control treatment. The culture establishment from the rhizome sprouted bud explant first appeared by root emergence from the explant (Figure 1A,B).

#### 2.1.2. Shoot Multiplication of ‘Bentong’ Ginger

##### Effects of Different Types of Cytokinins on Shoot Multiplication of ‘Bentong’ Ginger

All the cytokinin treatments in MS medium, including the control (cytokinin-free MS medium), proliferated multiple shoots of ‘Bentong’ ginger. The highest response of explants to multiple shoots induction was recorded in zeatin and BAP treatments, in which 100% of the explants produced multiple shoots (Table 2). The percentage of the responded explants was reduced with TDZ, kinetin, and control treatments, in which 93.33, 86.67, and 77.78% of the explants, respectively, responded to multiple shoots production. The shoot proliferation began with the emergence of new shoot primordia from the base of the main shoot. The emergence of the new shoot primordia was recorded almost at the same time in the treatments of zeatin and BAP after 6.87 and 7.13 days of culturing, respectively. In the treatments of kinetin and TDZ, shoots initiated significantly slower than the treatment of zeatin. The slowest response of explant to shoot initiation (after 10.83 days of culturing) was observed in the control treatment.

Besides the fast response, the highest number of shoots per explant (4.07 ± 0.12) was also observed in zeatin treatment. After zeatin, BAP produced more shoots per explants compared with that of TDZ and kinetin treatments. The lowest number of shoots per explant (1.93 ± 0.12) was recorded in the control treatment. The shoot length was not significantly affected by the cytokinin treatments, except for the treatment of TDZ, which resulted in the lowest shoot length (1.08 ± 0.07 cm) after six weeks of culture. The prolonged incubation in the treatment of TDZ caused a stunted growth of the shoots and was phytotoxic to the proliferated shoots.

In addition, the highest number of leaves per shoot (2.90 ± 0.19) was also observed in the treatment of zeatin. The number of leaves in BAP and kinetin treatment was not significantly different from the control treatment. The lowest number of leaves per shoot (1.20 ± 0.12) was produced in TDZ treatment. Root induction spontaneously occurred during shoot multiplication in all the treatments except for the treatment of TDZ. The highest number of roots per explant (9.43 ± 0.42) was observed in zeatin treatment. After zeatin, BAP and kinetin treatments induced more roots per explant, which were significantly higher than the control treatment.

##### Effects of Different Basal Media and Zeatin Concentrations on Shoot Multiplication of ‘Bentong’ Ginger

The analysis of variance showed that all measured parameters related to shoot proliferation and root induction were significantly affected at *p* < 0.01 by both factors of basal media and zeatin concentrations (Table 3). Linsmaier and Skoog (LS) [29] and MS media effectively enhanced shoot multiplication and their effects were not significantly different for all measured parameters. New shoots initiation started earlier with MS and LS media compared with Gamborg et al. [30] B5 medium. The number of shoots per explant, shoot length, and number of leaves per shoot were also significantly higher with MS and LS media compared with B5 medium. However, the number of roots per explant was considerably higher in the B5 medium than in MS and LS media.

The highest number of leaves per shoot was recorded in all media when supplemented with 10 µM of zeatin. However, the number of those leaves was not significantly different from zeatin-free media. In MS and LS media, a further increase of zeatin concentration of more than 10 µM caused a reduction in the number of leaves per shoot. In contrast to the number of shoots and leaves, the shoot length was reduced by adding zeatin to all basal media and a higher shoot length was recorded when zeatin was not added to the media. Roots were simultaneously induced in all the treatments. The addition of zeatin significantly increased the number of roots per explant in MS and B5 media compared with the zeatin-free medium. In LS medium, the number of roots was not significantly enhanced by the addition of zeatin. Although the B5 medium was not useful for the shoot multiplication of ‘Bentong’ ginger, it induced more roots per explant than MS and LS media.

Different zeatin concentrations also significantly affected all the recorded parameters, as presented in Table 3. The number of days to shoot initiation was gradually reduced from 13.33 ± 0.61 to 9.02 ± 0.14 by increasing the zeatin concentration from 0 to 20 µM. The lowest number of shoots per explant (1.98 ± 0.12) was induced in a zeatin-free medium and the highest number of shoots per explant (3.81 ± 0.17) was observed with the addition of 10 µM of zeatin. The highest number of leaves per shoot (2.8 ± 0.23) was also recorded with 10 µM of zeatin. A further increase of zeatin concentration of more than 10 µM decreased the number of shoots per explant and number leaves per shoot. The lowest number of leaves per shoot (2.23 ± 0.16) was recorded in 20 µM of zeatin. The longest shoot length (3.77 ± 0.1 cm) was recorded with zeatin-free media. The addition of zeatin in the culture medium significantly reduced shoot length compared with the zeatin-free medium. Besides, roots were simultaneously induced during shoot multiplication in all treatments of the zeatin and zeatin-free medium. The highest number of roots per explant (6.5 ± 0.29) was recorded in the culture medium supplemented with 5 µM zeatin. However, it was not significantly different with 10 and 15 µM of zeatin. Increasing zeatin concentration of more than 15 µM reduced the number of roots per explant. The lowest number of roots per explant was observed with the absence of zeatin in the media.

The interaction effects of different basal media and zeatin concentrations were only significant for the number of shoots per explant (Table 3). All basal media without zeatin supplementation resulted in a low number of shoots per explant. In MS and LS media, the number of shoots per explant was significantly increased proportionally to the concentration of zeatin from 0 to 10 µM (Table 4). The highest number of shoots per explant (4.28 ± 0.15) was recorded on MS medium supplemented with 10 µM of zeatin (Figure 1D). A further increase in zeatin concentration caused a reduction in the number of shoots per explant on both MS and LS media. Unlike MS and LS media, in B5 medium, the number of shoots per explant was gradually increased by increasing zeatin concentration from 0 to 20 µM.

#### 2.1.3. In Vitro Rooting of ‘Bentong’ Ginger

Roots were easily induced in ‘Bentong’ ginger and 100% of the explants responded to root induction in MS medium with all auxins and control (without auxin) treatments (Table 5). The effect of different types and concentrations of auxins on the number of days to the first root primordia emergence was not significantly different (*p* < 0.05) among all the treatments. Different types and concentrations of auxins caused a significant difference in the number of roots and root length of ‘Bentong’ ginger. The effects of different types of auxins on root induction were dependent on their concentrations. At a lower concentration of indole-3-acetic acid (IAA) and 1-naphthaleneacetic acid (NAA) (2.5 µM), the number of roots per plantlet was not significantly different from each other. However, at higher concentrations (5 or 7.5 µM), the number of roots per plantlet was significantly different from each other. Increasing the NAA concentration caused an increase in the number of roots per plantlet, and the highest number of roots per plantlet (15.44 ± 0.8) was induced with 7.5 µM of NAA (Figure 1F). Oppositely, increasing the IAA concentration reduced the number of roots per plantlet. Unlike NAA and IAA, no significant difference was observed in the number of roots with different concentrations of indole-3-butyric acid (IBA) in MS medium. The lowest number of roots per plantlet (6.33 ± 0.38) was recorded in the control treatment, but it was not significantly different from 7.5 µM IAA in the culture medium.

On the other hand, root length was increased in all types of auxin when increasing their concentration to 5 µM. The longest roots (3.94 ± 0.29 cm) were observed in the treatment of 5 µM IAA, but they were not significantly different from the treatments of 7.5 µM IAA and 5 µM IBA. Root length was reduced with treatments NAA compared with IAA and IBA. The shortest roots (1.93 ± 0.14 cm) were observed in the treatment of 2.5 µM NAA, but they were not significantly different with 5 and 7.5 µM NAA.

#### 2.1.4. Acclimatization of ‘Bentong’ Ginger In Vitro-Raised Plantlets

‘Bentong’ ginger in vitro-rooted plantlets (Figure 1F) were successfully acclimatized at a 95% survival rate in a growing media mixed of soil + coco peat + vermiculite (1:1:1 (*v*/*v*/*v*)) (Figure 1G). The acclimatized plantlets were successfully established (100%) in a shade house under a 50% black shade net (Figure 1H).

### 2.2. Quantification of Secondary Metabolites and Antioxidant Activity of Micropropagated and Conventionally Propagated Plant of ‘Bentong’ Ginger

#### 2.2.1. Total Phenolic Acid and Total Flavonoid Content

The results showed that the content of total phenolic acid, total flavonoid, and antioxidant activity of rhizome extract of micropropagated plants of ‘Bentong’ were not significantly different (*p* ≤ 0.05) from the rhizomes of conventionally propagated plants of ‘Bentong’ ginger (Table 6). Regardless of the propagation method, different types of solvents significantly affected the extracted amount of these secondary metabolites and antioxidant activity of micropropagated and conventionally propagated rhizome of ‘Bentong’ ginger. The highest phenolic acid content of both micropropagated (23.93 ± 0.48 mg GAE/g DW) and conventionally propagated rhizome of ‘Bentong’ ginger (23.1 ± 0.43 mg GAE/g DW) was obtained from the ethanolic extract. Acetone was the second-best solvent after ethanol for extracting the high quantity phenolic acid content. Hexane resulted in the lowest phenolic acid content in both the micropropagated (8.85 ± 0.52 mg GAE/g dry weight (DW) and conventionally propagated rhizome of ‘Bentong’ ginger (8.32 ± 0.63 mg GAE/g DW).

On the other hand, the highest flavonoid content of micropropagated (52.53 ± 2.01 mg RE/g DW) and conventionally propagated ‘Bentong’ ginger rhizome (51.75 ± 1.39 mg RE/g DW) was recorded in the acetone extract. After acetone, ethanolic extract contained a high content of flavonoids in both micropropagated and conventionally propagated ‘Bentong’ ginger rhizome. The lowest flavonoids content of micropropagated (16.08 ± 0.7 mg RE/g DW) and conventionally propagated rhizome (14.65 ± 0.39 mg RE/g DW) was recorded in the hexane extract. However, it was not significantly different from their aqueous extract.

#### 2.2.2. Antioxidant Activity

The results also showed that 2,2-diphenyl-1-picrylhydrazyl (DPPH) free radical scavenging activity and ferric reducing antioxidant power (FRAP) of ‘Bentong’ ginger rhizome was not significantly different at *p* ≤ 0.05 between micropropagated and conventionally propagated rhizome of ‘Bentong’ ginger (Table 7). However, different types of solvents with different polarities significantly affected the DPPH scavenging activity and FRAP of the ‘Bentong’ ginger rhizome extract. The highest DPPH scavenging activity of micropropagated (77.01 ± 0.83%) and conventionally propagated ‘Bentong’ ginger rhizome (75.46 ± 2.58%) was recorded in the ethanolic extract, and it was followed by acetone extract of both micropropagated and conventionally propagated rhizome of ‘Bentong’ ginger. The highest FRAP of micropropagated (64.10 ± 1.78 mg TE/g DW) and conventionally propagated rhizome (62.96 ± 0.67 mg TE/g DW) was also recorded in the ethanolic extract of ‘Bentong’ ginger rhizome. Water and hexane extracts of rhizome performed a lower DPPH scavenging activity and FRAP than the ethanolic and acetone extracts. This indicated that the extraction of the highest total phenolic acid content from the rhizome of ‘Bentong’ ginger positively affected its antioxidant activity of both micropropagated and conventionally propagated rhizome of ‘Bentong’ ginger.

## 3. Discussion

### 3.1. Micropropagation of ‘Bentong’ Ginger

‘Bentong’ ginger is an elite variety of *Zingiber officinale* in Malaysia, and it is less known about its micropropagation. This study’s approach was to establish a micropropagation protocol and evaluate the secondary metabolites content of the micropropagated and conventionally propagated plant of this variety. The rhizome sprouted buds were quickly responsive explant, but contamination-free culture establishment was a challenging task. A low concentration of Clorox was not effective in controlling bacterial contamination. Using 70% Clorox significantly reduced the contamination level, which resulted in 75% contamination-free explants. It is in confirmation with the findings of Azhar et al. [18] that more than 80% rhizome sprouted bud explants of *Boesenbergia rotunda* (Zingiberaceae) were aseptically established when they were double sterilized first with 60% Clorox for 30 min, followed by 20% Clorox for 15 min. Although 70% Clorox significantly reduced contamination of ginger rhizome sprouted bud explants, the aseptic explants’ survival rate was decreased in the culture owing to the damaging effect of a high concentration of Clorox. Hence, it is recommended to assess Clorox’s low concentration with a combination of some safe antibiotics.

Based on the shoot multiplication results, zeatin was found to be the best type of cytokinin for shoot multiplication of ‘Bentong’ ginger. The current study results are in confirmation with the findings of Rao et al. [31] that zeatin treatment produced a significantly higher number of shoots of *Alpinia galanga* (Zingiberaceae) compared with BAP, TDZ, and kinetin treatments. The significance of zeatin compared with BAP, kinetin, and TDZ has also been reported for shoot multiplication of several other herbaceous and non-herbaceous species [31,32,33,34]. Besides, zeatin was also found to be more effective for microrhizome induction of ‘Bentong’ ginger, but it was less effective for shoot multiplication compared with BAP [35]. The lower efficiency of zeatin for shoot multiplication compared with BAP could be due to the high oxidative cleavage of zeatin with a prolonged incubation in that study [36]. The effectiveness of zeatin in the present study could be due to its higher biological activity than the other types of cytokinins. The biological activity of the different cytokinins is different according to cytokinin receptors’ specificity to the structural variation of the cytokinin side chain or modification in the N7 and N9 position of the adenine ring [37]. The specific structure of zeatin with a double bond on the side chain between C2 and C3 and a hydroxyl group on C4 of the side chain contributes to its high biological activity [38]. Hence, in many bioassays, the association affinity of zeatin to cytokinin receptors was higher than the other isoprenoid and aromatic types of cytokinins [39,40]. This evidence supports the significance of zeatin for shoot multiplication of ‘Bentong’ ginger in the current study.

Different basal media effects showed that MS and LS media were more effective for shoot multiplication of ‘Bentong’ ginger than B5 medium. Bejoy et al. [41] also found that MS medium was more effective than B5 medium for shoot multiplication of *Curcuma vamana*. The diverse effects of different basal media could be due to the different ratios of the two nitrogen sources (ammonium and nitrate ions) in their composition [42]. Ivanova and Van Staden [43] evaluated the effects of ammonium and nitrate ions at different ratios on in vitro proliferation of *Aloe polyphylla,* and they observed a high rate of shoot proliferation with the application of equal proportions of ammonium and nitrate ions (30:30) or at the ratio of 20:40 and 40:20 in the culture medium. They indicated that a further increase of either ammonium or nitrate in the culture medium (10:50 or 50:10 ratio) caused a significant reduction in the shoots multiplication rate of *Aloe polyphylla*. This indicates that the balance of ammonium and nitrate ions (20.61:39.41) in MS and LS media could be the reason for their effectiveness in the present study. B5 medium containing a lower ratio (2.03:24.73) of ammonium and nitrate ions was not effective for shoot multiplication of ‘Bentong’ ginger. Apart from the ammonium and nitrate ratio, the total amount of nitrogen, sucrose, and macro and micronutrients, except potassium (K^+^) and sulfate (SO_4_^2−^) ions, are also higher in MS and LS media compared with that of B5 medium. The lower amount of sucrose (20 g L^−1^) in the B5 medium could also be a reason for the poor productivity of the shoot multiplication. The same efficiency of MS and LS media could be due to their same composition of macro and micronutrients and sucrose, except for the organic components.

The study results also showed that MS medium supplemented with 10 µM zeatin was the suitable culture medium for shoot multiplication of ‘Bentong’ ginger. Increasing the zeatin concentration in MS and LS media to more than 10 µM caused a reduction in the shoot multiplication rate. Similarly, a high level of zeatin of more than 9.12 µM reduced the number of shoots in other Zingiberaceae species, such as *Curcuma longa* L. [44], *Alpinia galanga* [31], and *Kaempferia galanga* L. [45]. The reduced shoot multiplication rate with a high level of zeatin could be due to the zeatin degradation by cytokinin oxidase or conversion of zeatin to its other biologically inactive or less active metabolites [46,47]. Exogenous application of cytokinin or the expression of isopentenyl transferase (IPT) gene increases the endogenous cytokinin level in the cell, which increases the cytokinin oxidase activity and, consequently, down-regulates the elevated cytokinin in the plant cells [48,49]. Hence, that could be the reason for the reduced shoot multiplication rate in MS and LS media with the application of the high concentration of zeatin in the current study. However, in B5 medium, increasing the zeatin concentration increased the shoot multiplication rate.

Theses interaction effects of basal media types and zeatin concentration could be due the different levels of ammonium in media composition. Ivanova and Van Staden [50] found that the multiplication rate of *Aloe polyphylla* was increased with the increasing zeatin concentration from 0 to 15 µM at the low concentration of ammonium (10.3 mM). Conversely, at high concentrations of ammonium (20.6 or 61.8 mM), the shoot multiplication rate was significantly reduced when 15 µM of zeatin was applied in the culture medium compared with 5 µM of zeatin. The level of ammonium form nitrogen in B5 medium (2.03 mM) is significantly lower than that of MS and LS media (20.6 mM). That could be the reason the number of shoots was increased in the B5 medium with the increasing zeatin concentration from 0 to 20 µM, whereas, in MS and LS media, the number of shoots was reduced with increasing the zeatin concentration to more than 10 µM.

Roots were spontaneously induced with the shoot multiplication stage of ‘Bentong’ ginger. This is in agreement with the previous reports that root induction occurred spontaneously with the shoot multiplication of ginger [19,21]. Although roots were spontaneously induced in all the treatments, these roots were not enough to transplant the plantlets in the case of separating the multiple shoots. An adequate number of roots per plantlet was required to transplant and survive the in vitro-raised ‘Bentong’ ginger plantlets. Therefore, a separate experiment was conducted to examine different types and concentrations of auxins for in vitro rooting of ‘Bentong’ ginger. The results showed that the treatment of 7.5 µM NAA was more effective for the induction of more roots per plantlet than IAA and IBA at similar concentrations. This is in agreement with the findings of Kambaska and Santilata [51] that NAA was more effective for in vitro root induction of ginger than IBA. Abbas et al. [26] also indicated that NAA was more effective for in vitro rooting of ginger than IAA. In conclusion, MS medium supplemented with 7.5 µM NAA was suitable for effective rooting of ‘Bentong’ ginger.

### 3.2. Quantification of Secondary Metabolites and Antioxidant Activity of Micropropagated and Conventionally Propagated Plant of ‘Bentong’ Ginger

The results of phytochemicals and antioxidant activities analysis showed that total phenolic acid, total flavonoid content, DPPH free radical scavenging activity, and FRAP of micropropagated ‘Bentong’ ginger rhizome were not significantly different from the conventionally propagated rhizome of ‘Bentong’ ginger. The current study results confirm the findings of Ma and Gang [52] that the secondary metabolites of micropropagated ginger rhizome were not significantly different from the conventionally propagated ginger rhizome. A slight increase in the secondary metabolites and antioxidant activities of micropropagated rhizome could be due to the effects of different plant growth regulators used during different stages of micropropagation of this plant [53]. Besides, micropropagated plants are encountered in a stressful environment during their transition from in vitro to ex vitro conditions. That stressful environment could also cause a slight increase in their secondary metabolites [54]. Regardless of the propagation method, different solvents with different polarities greatly influenced total phenolic acid and total flavonoid and the antioxidant activities of ‘Bentong’ ginger rhizome extract. Extraction with ethanol, a moderately polar solvent, resulted in the highest total phenolic acid, DPPH radical scavenging activity, and FRAP. This is in agreement with the findings of Tanweer et al. [55] that the highest total phenolic content of ginger rhizome was obtained from its ethanolic extract. Sharif and Bennett [56] also reported the highest total phenolic content and DPPH scavenging activity in the ethanolic extract of ginger rhizome. These findings indicate that ethanol’s polarity is more suitable for extracting ginger’s highest phenolic content, which contributes to its high antioxidant activity. However, the highest total flavonoid content was obtained from the acetone extract of the ‘Bentong’ ginger rhizome. Acetone was also reported for the highest total flavonoid content extraction of bottle gourd fruit [57]. From a toxicological point of view, ethanolic extract is safer than acetone and other organic solvents, and it has acceptability for human consumption. Therefore, ethanol is a suitable solvent for phenolic and flavonoid extraction of ‘Bentong’ ginger rhizome [58].

## 4. Materials and Methods

### 4.1. Micropropagation of ‘Bentong’ Ginger

#### 4.1.1. Plant Materials and Chemicals

Fresh mature rhizomes of ‘Bentong’ ginger were collected from Bentong, a western part of Pahang in Malaysia. All the chemicals required for basal media were analytical grade purchased from R & M Chemicals, Essex, UK. Zeatin, TDZ, and Gelrite were purchased from Duchefa, Haarlem, The Netherlands. Kinetin, BAP, NAA, IAA, and IBA were purchased from Sigma Chemical Company, Saint Louis, Missouri, USA.

#### 4.1.2. Explant Surface Sterilization and Culture Initiation

‘Bentong’ ginger rhizomes were kept in moist, sterilized sand in the dark at 25 ± 2 °C for three weeks to sprout the rhizome buds. The rhizome sprouted buds (1–1.5 cm) were excised from the rhizome and used as explants. The excised spouted buds were washed thoroughly for 30 min under running tap water with a few drops of liquid detergent and 2–4 drops of Tween 20. After washing, the rhizome sprouted bud explants were surface sterilized with 70% (*v*/*v*) ethanol for 1 min. Then, these explants were surface sterilized with different concentrations of Clorox (30, 40, 50, 60, and 70% (*v*/*v*)) with two drops of Tween 20 for 30 min immersion time. Explants’ surface sterilization with 70% ethanol for 1 min was considered as a control treatment. Finally, the surface-sterilized explants were washed 4–5 times with sterilized distilled water, and a few outer leaf sheets of the explants were removed. After that, the explants about 0.5–0.8 cm [21] were cultured in 35 mL vials containing MS medium supplemented with 30 g L^−1^ sucrose and solidified with 2.5 g L^−1^ gelrite. The pH of all media was adjusted to 5.8 using 0.1 N NaOH or HCl solutions before adding the gelrite. The media were heated in a microwave oven to homogenize the gelrite before dispensing into the culture vails. The culture vails were tightly closed by aluminium foil and then autoclaved at 121 °C and 104 kPa pressure for 20 min. Each treatment was replicated four times, with 20 explants per replication cultured in an individual vial. All cultures were incubated in the culture room at 25 ± 2 °C, under a 16-h photoperiod with a light intensity of 35 μmol m^−2^ s^−1^ provided by Philips cool white fluorescent tubes. Data related to the percentage of contamination-free culture and survival rate of the aseptic cultures were recorded after four weeks of inoculation.

### 4.1.3. In Vitro Shoot Multiplication

The contamination-free explants obtained from the culture initiation stage (Figure 1B) were cultured on MS medium supplemented with 10 µM BAP to provide enough explants for the shoot multiplication experiment. After four weeks, multiple shoots were produced and these shoots were used as explants for shoot multiplication. Before starting the experiment, the in vitro-raised shoots were cultured on MS hormone-free medium for two weeks to reduce the effects from the previously used BAP. After two weeks, the in vitro-raised shoots were shortened to 1 cm by maintaining the shoot base and then cultured onto the shoot multiplication medium (Figure 1C). Shoot multiplication of ‘Bentong’ ginger was studied by conducting two separate experiments. In the first experiment, MS medium supplemented with different types of cytokinin, BAP, kinetin, and zeatin at 10 µM and TDZ at 5 µM and MS cytokinin-free medium as control, were evaluated for shoot multiplication. Here, 10 µM of BAP, kinetin, and zeatin and 5 µM of TDZ were selected as the optimum concentration of these cytokinins from the previous research on ginger or other Zingiberaceae species [24,31,52]. Each treatment was replicated five times, with six explants per replication cultured in an individual flask (200 mL). After six weeks of inoculation, the data related to the percentage of explants responded to multiple shoots induction, number of days to shoots initiation, number of shoots per explant, shoot length (cm), number of leaves per shoot, and number of roots per explant were collected.

By determining zeatin as the best type of cytokinin, it was further studied with its different concentrations (0, 5, 10, 15, and 20 µM) with the combination of different basal media (MS, Gamborg et al. (B5) [30] and Linsmaier and Skoog (LS) [29]). Each treatment of this experiment was replicated three times, with six explants per replication cultured in an individual jar (300 mL) incubated in the same environmental conditions as mentioned above. After six weeks of inoculation, the optimum combination of the basal medium and zeatin concentration was selected based on the recorded data related to the number of days to shoot initiation, number of shoots per explant, shoot length (cm), number of leaves per shoot, and number of roots per explant.

### 4.1.4. In Vitro Rooting of ‘Bentong’ Ginger

In vitro-produced shoots of ‘Bentong’ ginger (3–4 cm) were used as explants in this experiment (Figure 1E). All the roots were trimmed out and cultured in 150 mL flask containing MS medium supplemented with different types of auxins, IAA, IBA, and NAA at 2.5, 5, and 7.5 µM, respectively, and MS auxin-free medium, were used as a control treatment. Each treatment was replicated three times, with six explants per replication. Data related to the number of days to initiate roots, number of roots per explant, and root length (cm) were collected after six weeks of culture.

### 4.1.5. Acclimatization and Ex Vitro Establishment of the In Vitro-Raised Plantlet

The in vitro-rooted plantlets were removed from the culture and washed thoroughly with running tap water to remove the media attached to the plantlet roots. The rooted plantlets were then planted in pots (200 mL) containing soil + coco peat + vermiculite (1:1:1) (Figure 1G). The pots planted with the in vitro-raised ‘Bentong’ ginger plantlets were kept for the first three weeks in the shade at 29 ± 2 °C and covered with transparent plastic bags. The plastic bags were punctured after one week and, after two weeks, they were removed entirely. The plantlets were kept for one week more in the same condition. After three weeks, the primary acclimatized plantlets were transferred to a shade house covered with a 50% black shade net. They were kept for three more weeks in the shade house at 33 ± 2/25 ± 2 °C day/night temperature. The plantlets were regularly irrigated in two-day intervals and, once a week, they were fertilized with 1/8 MS macro and micronutrient salts solution. The survival rate of the plantlets was recorded after three weeks in the shade house. Six-week-old tissue cultured raised seedlings, which were acclimatized in a growing media mixture of soil + coco peat + vermiculite (1:1:1), and pre-sprouted rhizomes of ‘Bentong’ ginger, were cultured in polybags (45 × 50 cm) filled with 15 L coco peat, burnt rice husk and chicken dung (5:5:1). These plants were maintained in a shade house under 50% black shade net and irrigated in two-day intervals. About 20 g NPK 12:12:17 + 2 MgO + TE fertilizer was used per plant two times after one and five months of transplanting.

## 4.2. Quantification of Secondary Metabolites Content and Antioxidant Activity of ‘Bentong’ Ginger

### 4.2.1. Planting Materials

The rhizomes of seven-month-old micropropagated and conventionally propagated ‘Bentong’ ginger plants were collected. The rhizomes were thoroughly washed and dried for 30 min inside the room. The rhizomes were sliced into small pieces (0.5 cm) and dried in the oven at 40 °C for 72 h. The dried rhizomes were powdered by a food blender, and the powder was kept in an airtight container and used for the evaluation of secondary metabolites and antioxidant activity.

### 4.2.2. Extraction of Antioxidant Compounds

The extraction of antioxidant compounds was carried out according to Hakiman and Maziah [59]. Briefly, 0.5 g of the rhizome’s powder was placed in the 150 mL conical flask. The conical flask was covered with aluminium foil, and 25 mL of each solvent such as distilled water, hexane, ethanol, and acetone was added into the conical flasks. The conical flask was placed on an orbital shaker (300 rpm) for an hour in the dark at 25 °C. The samples were filtered using Whatman No. 1 filter paper, and the extract was used for further analysis.

### 4.2.3. Chemicals and Reagents

The chemicals and reagents used in this study such as Folin–Ciocalteu reagent, sodium carbonate, sodium nitrite, aluminium chloride, sodium acetate, hydrochloric acid, acetic acid, sodium hydroxide, methanol, ethanol, acetone, and hexane were purchased from R & M Chemicals, Essex, UK. Gallic acid, rutin,2,2-diphenyl-1-picrylhydrazyl (DPPH), 6-hydroxy-2,5,7,8-tetramethylchroman-2-carboxylic acid (Trolox), and 2,4,6-tri (2-pyridyl)-s-triazine (TPTZ) were purchased from Sigma–Aldrich (St Louis, MO, USA).

### 4.2.4. Total Phenolic Acid Content

The total phenolic acid content assay was conducted according to the method reported by Haida and Hakiman [60]. The extracts at 0.5 mL volume and 4.5 mL of distilled water were added into test tubes. Then, 0.5 mL of Folin–Ciocalteu phenol reagent was added and mixed thoroughly by a vortex machine. After 5 min, 5 mL of 7% sodium carbonate was added. The final volume was adjusted to 12.5 mL by the addition of 2 mL of distilled water. The reaction mixtures were incubated for 90 min at room temperature. The absorbance of the reaction mixtures was measured at 750 nm wavelength using a UV–visible spectrophotometer (Thermo Fisher Scientific, Waltham, MA, USA). The extract’s total phenolic acid content was expressed as mg gallic acid equivalents per gram dry weight of the sample (mg GAE/g DW).

### 4.2.5. Total Flavonoid Content

The quantification of total flavonoid content was determined according to the method applied by Marinova et al. [55]. An amount of 0.5 mL of extracts was added into test tubes containing 2 mL of distilled water. Subsequently, 150 µL of 5% sodium nitrite was added and the mixture was incubated for 5 min, and then 150 µL of 10% aluminium chloride was added to the mixture. At the sixth minute, 1 mL of 1 M sodium hydroxide and 1.2 mL of distilled water were added. The mixture was mixed thoroughly and absorbance was measured at 510 nm wavelength using a UV–visible spectrophotometer. The total flavonoid content of extracts was expressed as mg rutin equivalents per gram dry weight of the sample (mg RE/g DW).

#### 4.2.6. 2,2-diphenyl-1-picrylhydrazyl (DPPH) Free Radical Scavenging Activity

2,2-diphenyl-1-picrylhydrazyl (DPPH) free radical scavenging activity of ‘Bentong’ ginger rhizome extract was measured by adding 40 µL rhizome extracted sample into 3 mL of 0.1 mM methanolic DPPH solution. The mixture was incubated at room temperature for 30 min and the absorbance was measured at 515 nm wavelength using a UV–visible spectrophotometer [61]. DPPH free radical-scavenging activity of the rhizome extracted sample was calculated using the following equation.

(1) $$\mathrm{DPPH}\;\mathrm{free}\;\mathrm{radical}\;\mathrm{scavenging}\;\mathrm{activity}\;(\%)\;=\;\frac{A_{1}-A_{2}}{A_{1}}\times 100,$$

where *A*_1_ is blank absorbance and *A*_2_ is sample absorbance.

#### 4.2.7. Ferric Reducing Antioxidant Power (FRAP) Assay

The ferric reducing capacity of ‘Bentong’ ginger rhizome extract was measured according to the method applied by Haida et al. [8]. Briefly, the FRAP reagent was prepared by mixing 300 mM acetate buffer (pH 3.6), 10 mM 2,4,6 tri(2-pyridyl)-s-triazine (TPTZ) prepared in 40 mM HCl, and 20 mM ferric chloride hexahydrate (FeCl3·6H2O) at a ratio of 10:1:1, respectively. Then, 1.5 mL of FRAP reagent was mixed with 100 µL of extract and the reaction mixture was incubated in the water bath at 37 °C for 30 min. The reducing power of the sample was measured at 593 nm wavelength using a UV–visible spectrophotometer. The FRAP value was expressed as mg trolox equivalent per gram dry weight of sample (mg TE/g DW).

### 4.3. Experimental Design and Statistical Analysis

A completely randomized design was applied for all the experiments. The data were analyzed by analysis of variance (ANOVA) using statistical analysis software (SAS) version 9.4 and means were separated by using Duncan’s multiple range test (DMRT) at *p* ≤ 0.05.

## 5. Conclusions

The study results showed that rhizome sprouted bud explants of ‘Bentong’ ginger were successfully established through in vitro culture by surface sterilization in 70% Clorox for an immersion duration of 30 min, which resulted in 75% aseptic culture. Shoot multiplication of ‘Bentong’ ginger was enhanced by optimizing the types and concentrations of cytokinins. Among different cytokinin types, zeatin performed better than BAP, kinetin, and TDZ for shoot multiplication of ‘Bentong’ ginger. By investigating different concentrations of zeatin, 10 µM of it added to MS medium positively influenced the shoot multiplication rate. MS medium supplemented with 7.5 µM NAA produced a sufficient number of roots per plantlet and these in vitro-rooted plantlets were successfully acclimatized in ex vitro conditions. After proper acclimatization, the in vitro-raised ‘Bentong’ ginger plantlets were successfully established with 100% in a shade house. The phytochemicals analysis showed that the total phenolic acid and total flavonoid and antioxidant activities of micropropagated plants ‘Bentong’ ginger were not significantly different from the conventionally plants of ‘Bentong’ ginger. Ethanol was the best solvent for extracting the phenolic compounds of both micropropagated and conventionally propagated rhizome of ‘Bentong’ ginger. This indicates that micropropagation of ‘Bentong’ ginger can be successfully adopted for large-scale production of disease-free planting materials of ‘Bentong’ ginger.

The combined application of cytokinins and auxins positively influenced the shoot multiplication of many plant species. Hence, further studies need to be conducted to obtain the best combination of cytokinins and auxin for shoot multiplication of ‘Bentong’ ginger. In addition, further studies are required to illustrate how different ammonium levels influence the effect of cytokinins on shoot multiplication. Moreover, it is recommended to evaluate the genetic fidelity of the in vitro-raised plantlets of ‘Bentong’ ginger with their mother plants. Besides rapid proliferation, tissue culture is a technique to improve the quality of the plant. Hence, it is recommended to test some elicitors for improving the secondary metabolites profile and antioxidant and activity of ‘Bentong’ ginger in vitro-raised plants.

## Figures and Tables

**Table 1:** Effects of different Clorox concentrations for 30 min immersion on explant surface sterilization and survival rate of ‘Bentong’ ginger rhizome sprouted buds explants.

Treatment (Clorox^®^ %)	Aseptic Culture ± SE (%)	Survival Rate ± SE (%)
Control	0.00 ± 0.00 e	0.00 ± 0.00 d
30	41.25 ± 3.75 d	100.00 ± 0.00 a
40	43.75 ± 3.15 cd	100.00 ± 0.00 a
50	51.25 ± 3.75 bc	100.00 ± 0.00 a
60	57.5 ± 3.23 b	91.48 ± 3.41 b
70	75 ± 3.54 a	83.60 ± 4.18 c

Values are means ± standard error (SE) (n = 80). Means within columns followed by the different letters are significantly different at *p* < 0.05 using Duncan’s multiple range test (DMRT).

**Table 2:** Effects of different types of cytokinins on the percentage of explants responded to multiple shoots induction, number of days to shoot initiation, number of shoots per explant, shoot length, number of leaves per shoot, and number of roots per explant of ‘Bentong’ ginger after six weeks of culture.

Cytokinin (µM)	Explant Responded to Shoot Induction (%)	Number of Days to Shoot Initiation	Number of Shoots/Explant	Shoots Length (cm)	Number of Leaves/Shoot	Number of Roots/Explant
Control (0)	77.78	10.83 ± 0.20 a	1.93 ± 0.12 e	3.73 ± 0.07 a	2.43 ± 0.12 b	4.07 ± 0.12 c
Zeatin (10)	100	6.87 ± 0.31 d	4.07 ± 0.12 a	3.71 ± 0.07 a	2.90 ± 0.19 a	9.43 ± 0.42 a
BAP (10)	100	7.13 ± 0.39 cd	3.53 ± 0.17 b	3.51 ± 0.15 a	2.45 ± 0.17 b	7.43 ± 0.31 b
Kinetin (10)	86.67	8.60 ± 0.27 b	2.40 ± 0.19 c	3.48 ± 0.35 a	2.05 ± 0.12 b	6.03 ± 0.37 b
TDZ (5)	93.33	8.00 ± 0.35 bc	3.07 ± 0.16 d	1.08 ± 0.07 b	1.20 ± 0.12 c	0.00 ± 0.00 d

Values are means ± standard error (n = 30). Means followed by the same letters in each column are not significantly different at *p* < 0.05 using Duncan’s multiple range test (DMRT). BAP = 6-benzylaminopurine and TDZ = thidiazuron.

**Table 3:** Main effects of different basal media and zeatin concentrations on number of days to shoot initiation, number of shoots per explant, shoot length, number of leaves per shoot, and number of roots per explant of Bentong ginger after six weeks of inoculation.

Treatment	Number of Days to Shoot Initiation	Number of Shoots/Explant	Shoot Length (cm)	Number of Leaves/Shoot	Number of Roots/Explant
Media					
MS	10.83 ± 0.40 b	3.35 ± 0.19 a	3.33 ± 0.11 a	2.80 ± 0.10 a	5.40 ± 0.20 b
LS	10.70 ± 0.36 b	3.15 ± 0.18 a	3.35 ± 0.11 a	2.88 ± 0.10 a	5.79 ± 0.20 b
B5	12.98 ± 0.62 a	2.90 ± 0.21 b	2.77 ± 0.14 b	1.87 ± 0.06 b	6.60 ± 0.34 a
Zeatin (µM)					
0	13.33 ± 0.61 a	1.98 ± 0.12 d	3.77 ± 0.10 a	2.49 ± 0.18 abc	4.59 ± 0.17 c
5	12.44 ± 0.54 ab	2.95 ± 0.10 c	3.00 ± 0.20 bc	2.67 ± 0.22 ab	6.50 ± 0.29 a
10	11.89 ± 0.61 b	3.81 ± 0.17 a	3.16 ± 0.12 b	2.80 ± 0.23 a	6.46 ± 0.30 ab
15	10.83 ± 0.46 c	3.58 ± 0.11 ab	3.07 ± 0.16 bc	2.39 ± 0.15 bc	6.28 ± 0.30 ab
20	9.02 ± 0.14 d	3.34 ± 0.13 b	2.76 ± 0.13 c	2.23 ± 0.16 c	5.81 ± 0.32 b
F value					
Media	25.99 ***	8.51 **	13.18 ***	50.22 ***	13.18 ***
Zeatin (µM)	26.26 ***	52.29 ***	10.59 ***	4.77 **	13.36 ***
Media × Zeatin (µM)	1.76 ^ns^	2.52 *	0.89 ^ns^	0.5 ^ns^	1.34 ^ns^
CV (%)	8.44	9.51	11.03	12.25	11.01

Values are means ± standard error (n = 18). Means followed by the same letters in each column are not significantly different at * *p* < 0.05 using Duncan’s multiple range test (DMRT). F value represented ** = *p* < 0.01, *** = *p* < 0.001, and ^ns^ = not significant. CV = coefficient of variation, MS = Murashige and Skoog medium, LS = Linsmaier and Skoog medium, and B5 = Gamborg et al. medium.

**Table 4:** Interaction effects of different basal media and zeatin concentrations on the number of shoots per explant of ‘Bentong’ ginger after six weeks of culture.

Zeatin (µM)	Basal Media		
	MS	LS	B5
0	2.22 ± 0.11 g	2.17 ± 0.17 g	1.56 ± 0.06 h
5	3.19 ± 0.10 cde	3.00 ± 0.19 ef	2.66 ± 0.09 fg
10	4.28 ± 0.15 a	3.90 ± 0.21 ab	3.27 ± 0.07 cde
15	3.73 ± 0.13 bc	3.57 ± 0.23 bcd	3.43 ± 0.23 b-e
20	3.31 ± 0.19 cde	3.12 ± 0.25 def	3.58 ± 0.22 bcd

Values are means ± standard error (n = 18). Means followed by the same letters are not significantly different at *p* < 0.05 using Duncan’s multiple range test (DMRT). MS = Murashige and Skoog medium, LS = Linsmaier and Skoog medium, and B5 = Gamborg et al. medium.

**Table 5:** Effect of different types and concentrations of auxin on rooting of in vitro-raised shoot of ’Bentong’ ginger after four weeks of the culture.

Treatment (Auxin)	Auxin Concentration (µM)	Plantlet Responded to Rooting (%)	Number of Days to Root Initiation	Number of Roots/Explant	Root Length (cm)
Control	0	100	6.06 ± 0.47 a	6.33 ± 0.38 e	3.16 ± 0.19 bc
IAA	2.5	100	5.89 ± 0.22 a	10.44 ± 0.62 bc	2.77 ± 0.21 bcd
IAA	5	100	5.44 ± 0.48 a	8.22 ± 0.59 de	3.94 ± 0.29 a
IAA	7.5	100	6.00 ± 0.19 a	6.67 ± 0.33 e	3.41 ± 0.29 ab
IBA	2.5	100	5.67 ± 0.51 a	7.22 ± 0.56 de	2.67 ± 0.24 cde
IBA	5	100	6.22 ± 0.48 a	9.11 ± 0.40 cd	3.35 ± 0.28 abc
IBA	7.5	100	6.33 ± 0.33 a	9.22 ± 0.48 cd	2.78 ± 0.2 bcd
NAA	2.5	100	6.00 ± 0.38 a	10.56 ± 0.78 bc	1.93 ± 0.14 f
NAA	5	100	6.11 ± 0.4 a	11.67 ± 1.02 b	2.03 ± 0.16 ef
NAA	7.5	100	6.78 ± 0.59 a	15.44 ± 0.80 a	2.19 ± 0.11 edf

Value are means ± standard error (n = 18). Means followed by different letters in each column are significantly different at *p* < 0.05 using Duncan’s multiple range test (DMRT). IAA = indole-3-acetic acid, NAA = 1-naphthaleneacetic acid, and IBA = indole-3-butyric acid.

**Table 6:** Total phenolic acid and flavonoid content of micropropagated and conventionally propagated rhizomes of ‘Bentong’ ginger.

Propagation	Solvent	Phenolic Acid (mg GAE/g DW)	Flavonoid (mg RE/g DW)
Conventional	Aqueous	16.53 ± 0.45 c	16.73 ± 0.53 cd
	Ethanol	23.1 ± 0.43 a	32.2 ± 0.64 b
	Acetone	19.85 ± 0.42 b	51.75 ± 1.39 a
	Hexane	8.32 ± 0.63 d	14.65 ± 0.39 d
	Mean	16.95	28.83
Micropropagation	Aqueous	16.64 ± 0.37 c	18.75 ± 0.45 c
	Ethanol	23.93 ± 0.48 a	33.74 ± 0.49 b
	Acetone	21.1 ± 0.43 b	52.53 ± 2.01 a
	Hexane	8.85 ± 0.52 d	16.08 ± 0.7 cd
	Mean	17.68	30.28
F value			
Propagation		4.16 ^ns^	4.30 ^ns^
Solvent		373.56 ***	595.03 ***
Propagation × Solvent		0.52 ^ns^	0.14 ^ns^
CV (%)		4.74	5.76

Values are means ± standard error (n = 12). Means followed by different letters in each column are significantly different at *p* < 0.05 using Duncan’s multiple range test (DMRT). F value represented *** = *p* < 0.001 and ns = not significant.

**Table 7:** 2,2-diphenyl-1-picrylhydrazyl (DPPH) free radical scavenging activity and ferric reducing antioxidant power (FRAP) assay of micropropagated and conventionally propagated rhizomes of ‘Bentong’ ginger.

Propagation	Solvent	DPPH Inhibition (%)	FRAP (mg TE/g DW)
Conventional	Aqueous	58.25 ± 0.01 c	40.39 ± 0.69 c
	Ethanol	75.46 ± 2.58 ab	62.96 ± 0.67 ab
	Acetone	71.61 ± 0.65 b	59.76 ± 1.17 b
	Hexane	61.56 ± 0.92 c	40.48 ± 2.07 c
	Mean	66.72	50.90
Micropropagation	Aqueous	63.32 ± 3.41 c	42.52 ± 0.64 c
	Ethanol	77.01 ± 0.83 a	64.10 ± 1.78 a
	Acetone	72.28 ± 1.16 ab	60.59 ± 1.16 ab
	Hexane	63.55 ± 0.25 c	43.37 ± 0.52 c
	Mean	69.04	52.65
F value			
Propagation		3.98 ^ns^	4.15 ^ns^
Solvent		40.69 ***	186.35 ***
Propagation × Solvent		0.68 ^ns^	0.3 ^ns^
CV (%)		4.2	4.06

Values are means ± standard error (n = 12). Means followed by different letters in each column are significantly different at *p* < 0.05 using Duncan’s multiple range test (DMRT). F value represented *** = *p* < 0.001 and ns = not significant.

## Data Availability

The data presented in this study are available in the article.
